# Pediatric Inhaled Medication: A Cross-Sectional Analysis on Usage Trends and Parental Perceptions in Romania

**DOI:** 10.3390/children12111436

**Published:** 2025-10-23

**Authors:** Alina Angelica Ciolpan, Mihai Craiu, Andreea Pușcașu, Mihai Alexandru Borcan, Valentina Daniela Comănici

**Affiliations:** 1 “Alessandrescu-Rusescu” National Institute for Mother and Child Health, Lacul Tei, Nr. 120, Sector 2, 020395 Bucharest, Romania; alina.angelica.ciolpan@gmail.com (A.A.C.); valentina.comanici@umfcd.ro (V.D.C.); 2 Faculty of Medicine, “Carol Davila” University of Medicine and Pharmacy, Bd. Eroii Sanitari 8, Sector 5, 050474 Bucharest, Romania; alexandru.borcan@stud.umfcd.ro; 3 Department of Pediatrics, Grigore Alexandrescu Clinical Emergency Hospital for Children, Bd. Iancu de Hunedoara, Nr. 30-32, Sector 1, 011743 Bucharest, Romania; andreea.puscasu0325@rez.umfcd.ro

**Keywords:** children, inhaled medication, parental perception

## Abstract

Background: Inhaled medications are central in managing pediatric respiratory diseases, yet device complexity and nebulizer use often challenge caregivers and affect adherence. This study assessed current usage patterns and parental perceptions of inhaled therapies in Romania, aiming to identify modifiable factors for targeted education. Methods: A cross-sectional observational survey was distributed via SVC^®^ (Spitalul Virtual pentru Copii—Virtual Children’s Hospital), a widely accessed social media platform for Romanian parents. Data included demographics, inhaled medication use, prescribing sources, adherence, and treatment perceptions. Results: A total of 1825 parents participated, mostly urban residents (87.6%). Chronic respiratory disease, mainly asthma, was reported in 7.3% of children. Inhaled therapy was used in 71.9% of cases, predominantly saline solutions, bronchodilators, and corticosteroids, marking a decline compared with the past decade. Nebulizers (74.1%) were the main devices used. Prescriptions were largely pediatrician-based, though self-medication occurred, particularly with saline solutions, often influenced by non-medical sources (*p =* 0.003). Parents of children with chronic disease were less likely to self-medicate (*p =* 0.042), especially with β_2_-agonists and corticosteroids (*p <* 0.001). Additionally, 31.3% reported use of complementary remedies, including homeopathy. Overall, 73.5% perceived inhaled therapy as effective, with adverse events reported by only 8.3%. Conclusions: Inhaled therapy remains prevalent in Romanian children, though self-medication and alternative treatments persist, shaped by cultural factors and limited medical education. Parents of children with chronic disease show better adherence and reliance on prescribed therapy. Enhancing parental education via accessible digital platforms may reduce inappropriate practices and improve outcomes in low-resource settings.

## 1. Introduction

Respiratory conditions represent a significant cause of morbidity and mortality among infants and children [1], chronic respiratory diseases showing a 39.5% increase from 1990 to 2017 [2]. Diseases affecting frequently pediatric population include respiratory conditions such as asthma, croup, bronchiolitis, and pneumonia [3], accounting for 25% of all pediatric medical consultations [4]. The cornerstone of drug delivery in prevalent respiratory diseases is inhaled medication therapy, which offers the benefits of rapid drug action onset, an enhanced therapeutic index, and diminished systemic side effects [5]. This method relies on a device capable of producing a cloud of small particles that remain airborne due to their low terminal settling velocity, allowing them to be inhaled and deposited in the airways. The primary mechanisms influencing the efficacy of inhaled medications are inertial impaction, gravitational sedimentation, and diffusion [6]. The anatomical and physiological differences in children can impact these three mechanisms by inconsistent breathing patterns, low tidal volumes, and high resistance due to the small cross-sectional diameter of the airways [7,8,9]. However, these devices are designed to reduce the accumulation of medication in oropharynx and deliver an appropriate dose to the lower respiratory pathway in a cost-effective and accessible approach [10].

The most common devices employed for the administration of inhaled medications include pressurized metered dose inhalers (pMDIs), dry powder inhalers (DPIs), and nebulizers [11]. While all these devices are easily accessible, they differ in the level of coordination required for effective use in children and tailoring approach is advisable [12]. pMDIs imply a high degree of coordination during inhalation to minimize particle deposition in oropharynx. For children under the age of six, pMDIs are typically used with a valved holding chamber or spacer, with a facemask in children younger than 3–4 years, or without, in older ones [13]. In contrast, DPIs require less coordination but rely on the patient’s inspiratory pressure to achieve an adequate flow rate of particles to the lungs, rendering them challenging for use in young children [14]. Nebulizers, on the other hand, do not depend on the child’s coordination, but request a longer duration for treatment and use of a facemask during the procedure [15,16]. The most frequent medication used with these devices includes inhaled bronchodilators and corticosteroids for asthma and recurrent wheezing, epinephrine for croup, and inhaled antibiotics, hypertonic saline solution, and mucolytic drugs for cystic fibrosis [17,18].

The diverse strategies of administration, the intricate technique involved, and low compliance rates among children make inhaled therapy a challenging procedure for caregivers [19,20]. In low-resource settings there is a prevalent tendency to administer off-label drugs during this procedure, a concern highlighted in another study conducted on the Romanian pediatric population [21]. The aim of our research was to document current trends and parental perception of inhaled medication usage in our country, to assess the vulnerabilities in medical care that could further be addressed.

## 2. Materials and Methods

### 2.1. Study Design

We conducted a cross-sectional, observational study utilizing an online questionnaire distributed via SVC^®^ (Spitalul Virtual pentru Copii—Virtual Children’s Hospital), a widely accessed social media platform for Romanian parents. The questionnaire was available for a limited period of 29 days, from 26 March 2025 to 23 April 2025 and targeted parents or legal guardians who had used at home inhaled medication for their children. We followed the STROBE statement in conducting the study and reporting the results.

### 2.2. Questionnaire Design

The questionnaire, titled “Exploring the Perception of Parents on Inhaled Medication in Children,” was piloted using Zoho Survey and comprised closed-ended, open-ended, and semi-structured questions. It consisted of 21 questions and we organized the results into five sections:

- Section 1 collected demographic data (including the child’s sex, urban or rural residence, and any previous diagnosis of chronic pulmonary disease);
- Section 2 gathered information on the history of inhaled medication usage in children. The initial question in Section 2 was, “Has your child ever received, or is currently receiving, inhaled medication at home?” Only an affirmative response granted participants access to subsequent questions, while a negative response directed them to Section 5;
- Section 3 addressed data about medical prescription and self-medication;
- Section 4 evaluated the parental perspective on the efficacy and side effects of inhaled medication;
- Section 5 collected data on other treatments used for respiratory conditions.

### 2.3. Data Collection

An invitation to participate was disseminated via SVC^®^. The invitation detailed the study’s objectives and included a link to an online questionnaire. Prior to accessing the questions, informed consent was obtained electronically from each respondent. Responses were collected anonymously to ensure patient confidentiality. The online questionnaire was available for 29 days, after which the data were recorded and managed using Microsoft Excel.

### 2.4. Statistical Analysis

We used JASP (Version 0.19.3) for data analysis. For qualitative variables, we used frequencies and percentages for a descriptive analysis of the data and Chi-square test to assess the relationship between them. In cases of expected counts below 5, Fisher’s Exact Test was applied to obtain more reliable p-values. Significance was measured at *p* = 0.05. The qualitative data were individually examined to discern recurring patterns and trends articulated by parents or legal guardians.

### 2.5. Ethical Considerations

This study received approval from the Ethics Committee of the National Institute for Mother and Child Health “Alessandrescu-Rusescu”, Bucharest, Romania, under approval number 21732/25 September 2024, in accordance with the Declaration of Helsinki. Parents or legal guardians were requested to provide electronic informed consent prior to responding to the questionnaire. Participation in this study was voluntary, and to ensure confidentiality, responses were collected anonymously.

## 3. Results

Out of 3723 individuals who accessed the online questionnaire, a total of 1825 participants provided responses. Among these, 103 individuals only partially completed the questionnaire, responding solely to Section 1. A total of 484 participants answered negatively to the initial question in Section 2, which averted them to Section 5, as progression through the other sections required an affirmative response. Conversely, 1238 respondents provided an affirmative answer, enabling them to proceed with the questionnaire. Among them, 22 abandoned the online questionnaire after Section 3 and 29 after Section 4. Ultimately, Section 5 was completed by 1553 respondents.

As the questionnaire was distributed online, participants had the autonomy to withdraw at any point, resulting in a variable number of responses for each section. Consequently, we will present the findings based on the number of responses received for each question.

### 3.1. Section 1—Demographic Data

Section 1 focused on the demographic characteristics and medical history of children, with an emphasis on chronic respiratory diseases. Among the 1825 responses, the majority of the children were female, comprising 75.67% of the sample, while males accounted for 24.32%, resulting in an approximate male-to-female ratio of 1:3. In terms of residence, 87.56% of participants resided in urban areas, whereas 12.43% were from rural areas, as shown in Table 1.

Concerning the medical history of respiratory diseases, only 7.34% of the children had been diagnosed with a chronic respiratory disease. Among these, asthma was the most prevalent condition, affecting 73.13% of the diagnosed children and 5.36% of all children. It was followed by recurrent wheezing (14.17%) and recurrent pneumonia (2.98%). Other conditions reported by parents included interstitial lung diseases, pulmonary atelectasis, bronchiectasis, recurrent bronchiolitis, recurrent bronchitis, broncho-pulmonary dysplasia, cystic fibrosis, and congenital cystic adenomatoid malformation.

### 3.2. Section 2—History of Inhaled Medication Usage in Children

Participants were permitted to proceed with Sections 2, 3, and 4 only if they provided an affirmative response to the initial question of Section 2 (“Has your child ever received, or is currently receiving, inhaled medication at home?”). In the absence of such a response, respondents were directed to Section 5. Of the 1825 respondents from Section 1, 1722 answered the first question, resulting in 484 participants being redirected to the final question, while 1238 were allowed to continue with the subsequent sections.

Upon analysis, it was determined that 71.89% (*n* = 1238) of the children had received inhaled medication at least once in their lifetime. The treatments were primarily administered once or twice annually (*n* = 559, 45.15%). Less frequent was the administration of inhaled medication five to six times per year, observed in 8.88% of cases, while monthly administration was noted in only 3.23% of cases as shown in Table 2.

Regarding the types of medications administered for inhalation therapy, the multiple-response question revealed that the majority of children received saline solutions (*n* = 940, 75.92%). Other inhaled treatments included inhaled bronchodilators (*n* = 652, 52.66%), inhaled corticosteroids (*n* = 479, 38.69%), and nebulized epinephrine (*n* = 281, 22.69%). A smaller proportion of children were treated with dexamethasone, acetylcysteine and hyaluronic acid.

The aforementioned inhaled medication was predominantly delivered using a Nebulizer (*n* = 918, 74.15%), followed by a Metered Dose Inhaler (MDI) (*n* = 247, 19.95%), Dry Powder Inhaler (DPI) Turbuhaler (*n* = 69, 5.57%), and DPI Diskus (*n* = 4, 0.32%). On this question only 1238 provided responses. Regarding the type of nebulizer employed for administering inhaled medication, 825 out of 918 nebulizer users responded to this question. The Ultrasonic Nebulizer was preferred (*n* = 383, 46.42%), followed by the Jet Nebulizer (*n* = 373, 45.21%) and the Mesh Nebulizer (*n* = 69, 8.36%).

### 3.3. Section 3—Medical Prescription of Treatment and Self-Medication

In this section, we sought to examine whether the administration of inhaled medication to children was consistently guided by medical professionals. In a multiple answer question with 1238 respondents, in the majority of instances, the inhaled treatment was recommended by a pediatrician (*n* = 772, 62.35%), followed by a pulmonologist (*n* = 323, 26.09%), a general practitioner (*n* = 93, 7.51%), an otolaryngologist (*n* = 29, 2.34%), and an allergist (*n* = 16, 1.29%). Nonetheless, a small proportion of parents or legal guardians independently opted for this treatment or relied on advice from online sources, family, or friends (*n* = 35, 2.82%), as shown in Table 3.

Of the 1216 respondents, 876 (72.03%) received a medical recommendation for every instance of inhaled therapy usage. However, 27.38% of parents or legal guardians reported using this type of treatment partially on medical advice and partially independently. Only 0.57% chose inhaled medication without any medical recommendation.

### 3.4. Section 4—Evaluating Perception on Efficacy and Side Effects of Inhaled Medication

In assessing perceptions regarding the efficacy and side effects of inhaled medication, data from 1187 respondents revealed that 873 individuals (73.54%) reported an improvement in children’s medical conditions. Conversely, 279 respondents (23.50%) expressed partial satisfaction, and 35 respondents (2.94%) indicated no health improvement, as shown in Table 4.

Concerning side effects, the majority from 1187 respondents (*n* = 1088, 91.65%) did not report any issues associated with the use of inhaled medication. Among the remaining 99 respondents (8.34%), complaints included tachycardia (36.36%), agitation (26.26%), skin rash (18.18%), persistent cough (12.12%), and less frequently, dysphonia, tremor, growth impairment, oral candidiasis, pain, pneumonia, and headache.

### 3.5. Section 5—Other Treatments for Respiratory Disease

In Section 5, we received answers from both participants who use and do not use inhaled medication. In case of respiratory distress in children, they received alternative medication in a percentage of 31.29% (*n* = 486), as shown in Table 5. They mentioned natural remedies and homeopathy (*n* = 240, 49.38%), air humidification (*n* = 211, 43.41%), nasal sprays (*n* = 173, 35.59%), antihistamine treatment (*n* = 37, 7.61%), antibiotics (*n* = 30, 6.17%), non-steroidal anti-inflammatory drugs (*n* = 29, 5.96%) and, in small percentages, montelukast, N-acetylcysteine and oral immuno-modulators.

### 3.6. Section 6—Statistical Analysis

Examining demographic data, the chi-square test revealed a significant association between sex and the incidence of chronic pulmonary disease (*p <* 0.001), with a higher prevalence observed in the male population (13.28% vs. 5.43%). No statistically significant association was found between residence and the presence of chronic pulmonary disease in children (*p =* 0.71). Furthermore, the relationship between urban or rural residency and the use of inhaled medication at home was not statistically significant (*p =* 0.48). We found an association between the presence of chronic respiratory disease and the history of inhaled medication (*p <* 0.001), with a percentage of 96.80% of children with chronic respiratory conditions receiving inhaled treatment compared to only 69.94% in those without a chronic disease.

In assessing the relationship between the type of inhaled medication used and the source of information about treatment, the chi-square test was employed or Fisher’s exact test when the frequency was less than 5. Given that the question concerning the source of treatment recommendations permitted multiple responses, we categorized the participants into two distinct groups: those who utilized at least once non-medical source for administering inhaled treatment to their child, and those who relied solely on medical prescriptions. There were no statistically significant associations between medical prescription and the use of inhaled bronchodilators (*p =* 0.062) or inhaled corticosteroids (*p =* 0.051). Conversely, the use of saline solutions was more prevalent among parents informed by family, friends, or online sources rather than medical sources (*p =* 0.003, 97.14% vs. 75.31%). Also, there was no significant association between the source of recommendation and inhaled epinephrine administration (*p =* 0.4).

Conducting a chi-square test on the relationship between the source of recommendation for inhaled medication and the perceived effect of the treatment, no statistically significant association was found between the medical and non-medical sources of documentation (*p =* 0.546). Similarly, there is no statistical association between the source of treatment recommendation and reported side effects (*p =* 0.151).

Conducting Fisher’s exact test to examine the association between the diagnosis of chronic respiratory disease and the source of inhaled medication recommendation, a significant association was identified (*p =* 0.042). Our data indicates that medical advice for inhaled treatment was received by 100% of children with a chronic respiratory disease, compared to 96.86% of those without a known respiratory disease. Furthermore, a history of respiratory conditions was associated with a higher prevalence of receiving inhaled beta β_2_-agonists (*p <* 0.001) and corticosteroids (*p <* 0.001). Conversely, children without chronic pathology were more frequently administered nebulized epinephrine (*p =* 0.004) and saline solutions (*p <* 0.001).

## 4. Discussion

This relevant topic is backed up by existing studies that indicate a prevalent phenomenon in Romania, where parents often self-medicate their children [21,22]. The research conducted by Tarciuc et al. revealed that 70% of caregivers in Romania administer medication to their children without medical advice [23]. Our research specifically focuses on inhaled medications, a particularly sensitive subject due to several aspects: limited parental knowledge regarding this form of treatment, very frequent use, high variability of available devices across pediatric age span [24,25,26,27].

Our study concluded that in Romania, 71.89% of children have a history of using inhaled medications at home, with 62.35% of these prescriptions originating from pediatricians. These findings align with research conducted in France, where 83.1% of children received respiratory medications, with 63% of prescriptions from general practitioners [28]. Furthermore, among children with chronic respiratory diseases, asthma was the most prevalent condition in which inhaled medicine was used (9.8% compared to 5.36% in our study, probably due to lower prevalence of asthma in Romania compared with France) [29]. Differences were observed in the types of medications used. In the study by Benevent et al. [28], the majority of children received inhaled corticosteroids (95.3%), followed by inhaled short-acting β_2_-agonists (68.8%), whereas in our study, these medications were used less frequent, 38.69% and 52.66%, respectively. In contrast, Romanian caregivers predominantly used saline solutions, with a substantial usage rate of 75.92%. The unchallenging effort for this medication over the counter procurement may account for its prevalent usage patterns, beside perceived innocuity. Our research corroborates these findings, indicating that the use of saline solution is often associated with non-medical sources, such as family, friends, or online information (*p =* 0.003). Although only 2.82% of caregivers reported to follow non-medical advice, there is a noticeable trend within the Romanian population to use inhaled medication partially on self-medication (27.38%). This suggests that following a medical consultation for a respiratory condition, parents may inaccurately replicate the same treatment for subsequent respiratory diseases that share some of the clinical features present in previous episodes. This could be explained by another study focusing on the Romanian population which attributes this self-medication trend to the scarcity of healthcare services and limited health education, adding that some pharmacists easily dispense prescription drugs [30].

Potential benefits of inhaled hypertonic saline solution include reducing duration of hospital stay, alleviation of symptoms, and decrease in admission rates in children with acute bronchiolitis and acute wheezing episodes [31,32]. This mediation also has a substantial contribution to cystic fibrosis treatment [33]. Hypertonic saline solution use without medical prescription poses potential risks that seem to be disregarded by most of our respondents. Notable side effects include sore throat, shortness of breath, spastic cough, and bronchospasm. The use of hypertonic saline solutions can increase production of bronchial secretion, which is particularly unsafe for young children who may lack the ability to effectively expel sputum, thereby increasing the risk of bronchospasm or lower respiratory tract infections [34].

Moreover, among the caregivers surveyed, 31.29% reported to use alternative treatments for children’s respiratory conditions. Notably, 49.38% indicated the use of natural remedies and homeopathy, a result that aligns with previous studies on the Romanian population, where 59% of caregivers employed medicinal plants treatment [35] and 31.84% of parents used homeopathic remedies [36]. While research has demonstrated the beneficial effects for certain herbal medicines [37], such support is lacking for homeopathic treatments in managing or preventing acute respiratory tract infections that trigger exacerbations in asthmatic children [38].

Evaluating the effectiveness of the inhaled medication, the majority of parents or legal guardians assessed the treatment as effective (73.54%), whereas a mere 2.94% considered it completely ineffective. This finding contrasts with other studies on parental perceptions of inhaled therapy in children, where a significant proportion of caregivers expressed concerns and reservations regarding this type of treatment [39,40]. Furthermore, no significant association was identified between the source of the medication and the perceived efficacy (*p =* 0.546). The source of recommendation does not appear to influence the perception of treatment efficacy in our study. This may be attributed to the more frequent use of saline solutions among parents informed by non-medical sources (*p =* 0.03), a medication known for its efficacy in various respiratory conditions [41].

Analyzing the group of children diagnosed with chronic respiratory disease, it was observed that they are more likely to use inhaled medication (*p <* 0.001), adhere more frequently to medical recommendations for inhaled treatment (*p =* 0.042), and have a higher prevalence of receiving inhaled β_2_-agonists (*p <* 0.001) and corticosteroids (*p <* 0.001). These findings characterize children from Romania with a history of chronic respiratory disease as more medically compliant, exhibiting increased trust in healthcare providers and medical treatments. This may be attributed to the increased sense of responsibility demonstrated by parents of children with chronic respiratory conditions, which fosters increased vigilance and stronger adherence to prescribed medical regimens [42].

To the best of our knowledge, this investigation constitutes the largest national study to date exploring parental perceptions regarding inhaled medication. The principal limitation of this research lies in its self-reported, cross-sectional, online design, which may introduce selection and recall biases and consequently restrict the generalizability of the findings. Despite the substantial volume of data collected, an additional limitation stems from the initial questionnaire design, which resulted in the loss of potential responses from 484 participants (28.1%). These individuals provided a negative response to the initial item in Section 2, which directed them to Section 5, as continuation through the subsequent sections required an affirmative answer. Another potential limitation of our study is related to parental understanding of some of the medical terms used in the questionnaire, aspect that could be responsible for reporting bias.

Future studies could replicate the present work to assess the impact of ongoing awareness and educational initiatives disseminated through social media platforms—currently the most frequently accessed sources of information by parents.

## 5. Conclusions

Inhaled therapy adequacy, adherence and compliance are improving in the last decade for Romanian children, though self-medication and alternative treatments persist, shaped by cultural factors and limited medical education. Enhancing parental education via accessible digital platforms, like SVC^®^, may reduce inappropriate practices and improve outcomes in low-resource settings.

At present parents of children with chronic disease from Romania show better adherence and reliance on prescribed therapy compared with previous publications and can be empowered towards active implication in their children’s treatment via social media tools.

## Figures and Tables

**Table 1:** Demographic data of pediatric population included in the study.

	*n*	%	Total Responses
Sex			1825
Female	1381	75.67	
Male	444	24.32	
Residence			
Urban	1598	87.56	
Rural	227	12.43	
History of chronic respiratory disease			
No	1691	92.65	
Yes	134	7.34	
Asthma	98	73.13	134
Recurrent wheezing	19	14.17	
Recurrent pneumonia	4	2.98	
Cystic fibrosis	3	2.23	
Broncho-pulmonary dysplasia	2	1.49	
Bronchiectasis	2	1.49	
Others	6	4.47	

**Table 2:** History of Inhaled Medication Use in Study Participants: Medication Types and Administration Methods.

	*n*	%	Total Responses
History of inhaled medication usage			1722
Yes	1238	71.89	
No	484	28.1	
Administration Frequency			1238
Single administration (once in a lifetime)	147	11.87	
Infrequent administration (<1 time per year)	53	4.28	
Occasional administration (1–2 times per year)	559	45.15	
Intermittent administration (3–4 times per year)	239	19.3	
Frequent administration (5–6 times per year)	110	8.88	
Regular administration (monthly)	40	3.23	
Chronic use (>3 months)	90	7.26	
Medication Classes Used in Inhalation Therapy *			
Saline solutions	940	75.92	
Inhaled bronchodilators	652	52.66	
Inhaled corticosteroids	479	38.69	
Nebulized epinephrine	281	22.69	
Dexamethasone	39	3.15	
Acetylcysteine	20	1.61	
Hyaluronic acid	7	0.56	
Methods of inhaled medication delivery			
Metered Dose Inhaler (MDI)	247	19.95	
Dry Powder Inhaler (DPI) Turbuhaler	69	5.57	
DPI Diskus	4	0.32	
Nebulizer	918	74.15	
Ultrasonic Nebulizer	383	46.42	825
Jet Nebulizer	373	45.21	
Mesh Nebulizer	69	8.36	

* Questions allowing multiple answers were indicated with an asterisk.

**Table 3:** Use of inhaled medication: prescribed vs. non-prescribed.

	*n*	%	Total Responses
Source of treatment recommendation *			1238
Pediatrician	772	62.35	
Pulmonologist	323	26.09	
General Practitioner	93	7.51	
Otolaryngologist	29	2.34	
Allergist	16	1.29	
Online sources, family, or friends	35	2.82	
Physician prescription or self-medication			1216
Use exclusively under medical recommendation	876	72.03	
Partially medical advice, partially self-medication	333	27.38	
Only self-medication	7	0.57	

* Questions allowing multiple answers were indicated with an asterisk.

**Table 4:** Parental Perception of Inhaled Medication Efficacy and Side Effects.

	*n*	%	Total Responses
Perception on efficacy of inhaled medication			1187
Effective	873	73.54	
Partially effective	279	23.50	
Ineffective	35	2.94	
Side effects			
Not reported	1088	91.65	
Reported *	99	8.34	
Tachycardia	36	36.36	99
Agitation	26	26.26	
Skin rash	18	18.18	
Persistent cough	12	12.12	
Dysphonia	5	5.05	
Tremor	5	5.05	
Growth impairment	5	5.05	
Oral candidiasis	4	4.04	
Pain	4	4.04	
Pneumonia	3	3.03	
Headache	1	1.01	

* Questions allowing multiple answers were indicated with an asterisk.

**Table 5:** Other treatments used for respiratory disease.

	*n*	%	Total Responses
Other treatments for respiratory disease			1553
No	1067	68.70	
Yes *	486	31.29	
Natural remedies and homeopathy	240	49.38	486
Air humidification	211	43.41	
Nasal sprays	173	35.59	
Antihistamine treatment	37	7.61	
Antibiotics	30	6.17	
Non-steroidal anti-inflammatory drugs	29	5.96	
Montelukast	18	3.70	

* Questions allowing multiple answers were indicated with an asterisk.

## Data Availability

The data presented in this study are available on request from the corresponding author. The data are not publicly available due to restrictions, e. g., privacy or ethical.

## Abbreviations

The following abbreviations are used in this manuscript:

SVC	Spitalul Virtual pentru Copii—Virtual Children’s Hospital
pMDIs	Pressurized metered dose inhalers
DPIs	Dry powder inhalers
STROBE	Strengthening the Reporting of Observational Studies in Epidemiology
